# Digitization of hospital administration and public service reform: integration of technology and humanistic values

**DOI:** 10.3389/fpubh.2025.1743085

**Published:** 2026-01-13

**Authors:** Endah Woro Utami, Choirul Shaleh, Endah Setyowati, Hermawan Hermawan, H. T. Amir, Irtanto Irtanto, Andjar Prasetyo

**Affiliations:** 1 Faculty of Administrative Sciences, Brawijaya University, Malang, East Java Province, Indonesia; 2 Regional Research and Innovation Agency of East Java Province, Surabaya, East Java Province, Indonesia; 3 Bappeda Kota Magelang, Magelang, Central Java, Indonesia

**Keywords:** digital governance, digital inclusion, digital transformation, e-government, public services, quality of health services, regional hospitals, service efficiency

## Abstract

This study aims to analyze the implementation of digital governance in improving the quality of public health services in the Ngudi Waluyo Wlingi Regional General Hospital Rumas, Blitar, East Java Province, Indonesia. This study uses a qualitative approach with a case study design, involving data collection techniques in the form of in-depth interviews with 28 primary informants, participatory observation, and document analysis. The findings show that digitalization and national platforms such as SatuSehat have improved service efficiency, transparency, and responsiveness. The principles of digital governance—user need, privacy and security, interoperability, and inclusion—have been implemented adaptively, although they still face inclusivity challenges for older adults patients and Social Security participants. The theoretical implications are to strengthen the digital governance framework in the context of public health services, while practically providing a responsive and participatory digital governance model for regional hospitals. This study makes a unique contribution through an integrative approach among digital technology and humanistic values in healthcare.

## 1 Introduction

Digital transformation in the health sector has become a strategic priority in improving the efficiency, transparency, and quality of hospital services, especially in the post-pandemic era which demands a fast, accurate, and integrated service system. Regional hospitals such as Ngudi Waluyo Hospital need digital innovations that are not only technologically modern, but also able to answer the needs of patients and medical personnel directly. Digitalization, if carried out with good governance, has the potential to accelerate equitable distribution of health services and increase patient satisfaction through more responsive and secure services. However, the implementation of digitalization in hospitals still faces various crucial problems. Obstacles such as system interoperability, limited infrastructure, lack of digital skills of medical personnel, and low user participation are major barriers in ensuring the success of digital transformation (1–3). In addition, there are not many comprehensive evaluation frameworks that can be used to systematically assess the extent to which digitalization contributes to improving the quality of services in hospitals, especially in the context of regional hospitals.

Previous research has emphasized partial aspects of digital transformation such as the technical aspects of information systems or regulatory challenges, but has not fully incorporated digital governance, patient experience, and quality of public services in one structured evaluative approach. This creates a knowledge gap in understanding how digitalization has a real impact on improving the overall quality of health services in the regional hospital environment. Based on this, this study aims to develop and implement the Healthcare Digital Transformation Evaluation Framework that integrates the SERVQUAL model from Parasuraman, the digital governance principles of the Department of Health and Social Care UK, and the concept of public service quality from Milakovich. This framework will be tested at Ngudi Waluyo Hospital as a case study, to make an empirical and practical contribution in evaluating the effectiveness of the hospital’s digital transformation as a whole and based on local needs.

Digital governance plays a crucial role in digital transformation in the healthcare sector to improve the efficiency, transparency, and quality of hospital services. Fernandes et al. (2) found that effective governance, strong leadership, and regulation are clearly key to the success of digitalization, although there are still obstacles such as policy fragmentation and system interoperability. Jaana et al. (3) emphasized the importance of active participation of medical personnel, staff training, and sustainable funding support in ensuring the sustainability of e-health innovations such as the Cardiac Virtual Care program. Dal Mas et al. (1) identified multidimensional challenges in digital transformation, including system interoperability, organizational resilience, lack of digital human resources, and regulatory uncertainty. Mattei (4) added that the slow adoption of technology in the European health system is caused by bureaucratic resistance, limited skills of medical personnel in using artificial intelligence (AI), and regulations that have not been adaptive. Visconti and Morea (5) show that digitalization can improve cost efficiency through IoMT and big data, with P4P schemes as a fair funding model, while Moro Visconti and Martiniello (6) highlight the potential of smart hospitals in improving service quality through IoT and AI, but require public-private partnerships and results-based financing models. Masuda et al. (7) found that Electronic Health Records (EHR) still faces issues of interoperability and data security, so a framework such as AIDAF is needed to support system integration, while Ikawati (8) proves that Electronic Medical Records (RME) improves the coordination of medical teams and reduces recording errors, but its implementation is hampered by data security issues and staff training. At the regional hospital level, Gunawan et al. (9) evaluated information technology (IT) governance using the COBIT framework and found that the lack of attention to the IT division hampered the performance of healthcare services, while Odelia (10) found that the implementation of Hospital Management Information Systems (HMIS) at Dr. Mohamad Soewandhie Hospital Surabaya increased organizational capacity, but was still constrained by inadequate network infrastructure and hardware. Overall, all studies agree that the success of digital transformation in hospitals requires a combination of good governance, institutional support, adequate infrastructure, and holistic integration of digital policies and strategies, especially in the context of regional hospitals such as Ngudi Waluyo Hospital.

## 2 Literature review

Public administration as a social science studies the process of formulating and implementing public policies through the interaction of the state and society to fulfill the public interest, with scopes such as digital-based public policy, management, and public services. The paradigm of public administration is now shifting toward citizen-centric digital governance that emphasizes public engagement, transparency, and service transformation (11). In the context of healthcare, the implementation of a quality-of-service framework such as SERVQUAL has proven relevant for measuring quality gaps and user expectations (12). Regional hospitals such as Ngudi Waluyo Hospital are at the forefront of implementing digital governance to improve efficiency, transparency, and create public value through inclusive and responsive health services (13). Meanwhile, the quality of health services is an important indicator that includes medical outcomes, patient experience, efficiency, and accountability of service providers. The Donabedian model remains relevant for evaluating quality through structural, process, and outcome dimensions, while the SERVQUAL model enriches measurements with five dimensions such as reliability and empathy, although it needs to be adapted to the context of modern healthcare (14). Recent studies have also shown the need for multidimensional approaches and contextual measurement models such as HEALTHQUAL and SERVPERF to better align quality evaluations with patient needs and local conditions (15, 16).

Digital governance is a governance approach that utilizes digital technology to increase transparency, efficiency, participation, and responsiveness of public services, as well as encourage collaboration among the government, the community, and the private sector so that more data-based and inclusive services are created (17). Different from just administrative digitalization, digital governance emphasizes structural.

The implementation of digital governance in the health sector increases efficiency, transparency, service quality, and public participation through technologies such as RME and HMIS, while strengthening collaborative relationships among the government and citizens (17). This holistic approach helps ensure digitalization not only becomes technical modernization, but also supports social justice and active participation in healthcare.

## 3 Methods

This study uses a qualitative design (18) with an interpretive case study approach to deeply understand the improvement of the quality of health services in the perspective of digital governance at the Inpatient Installation of Ngudi Waluyo Wlingi Hospital, Blitar Regency, East Java Province, Indonesia. This design was chosen to explore the social realities and perceptions of key actors in a contextual and holistic manner, thereby allowing researchers to capture the dynamics of digital health service policy implementation. The sample of this study was determined by a purposive sampling technique that considers informants to have a deep understanding of digital governance practices and policies at Ngudi Waluyo Hospital. There were a total of 28 informants consisting of hospital staff (structural and functional positions) and patients/visitors as service recipients. The sample selection is flexible and develops until the data saturation point is reached. In addition to purposive sampling, snowball sampling techniques are used to recommend more relevant informants, ensuring completeness and depth of data. Primary data were obtained through semi-structured in-depth interviews using interview guidelines compiled based on the SERVQUAL theoretical framework, DHSC principles, and digital governance indicators (efficiency, transparency, quality of service, public participation). The interviews were conducted for 5 months, starting from September 16, 2024 to February 16, 2025, by ethics code No. B/070/DIKLAT/2499/409.52.4/2025 from Ngudi Waluyo Wlingi Hospital. In addition to interviews, direct participatory observations were conducted to observe the implementation of systems such as RME, HMIS, and telemedicine in the field. Secondary data was obtained from the review of official documents such as Law Number 17/2023 on Health, Presidential Regulation No. 95/2018 on Electronic-Based Government Systems, hospital service guidelines, as well as internal reports and blueprints for hospital digital transformation.

The analysis process follows the Tracy’s (19) model which includes three stages: Data condensation through selection, focusing, and simplification of data from interviews, observations, and documents to answer the research focus. Data presentation with visualizations in the form of tables, diagrams, and narratives to facilitate interpretation. Drawing conclusions and verifying by identifying patterns, relationships, and factors that support/inhibit service quality improvement, which are then verified to maintain the validity of the results. Validity is maintained through triangulation of sources and methods, comparing data from informants, observations, and documents. Continuous observations for 5 months were carried out to ensure data consistency. The transferability is strengthened by a detailed and authentic description of the research context. The dependability and confirmability aspects are maintained through trial audits, namely recording the details of the data collection and analysis process, so that research steps can be traced and replicated. With this method design, the research not only meets strict scientific standards but also provides a comprehensive and accountable understanding of the apply digital governance in improving the quality of health services at Ngudi Waluyo Wlingi Hospital.

## 4 Results

This research focuses on digital governance at Ngudi Waluyo Wlingi Hospital, Blitar, which is located at Jl. Dr. Soecipto No. 5, Wlingi, Blitar Regency, East Java Province, Indonesia. This hospital, with an area of 26,490.69 m^2^, is a Regional Public Service Agency whose operations are regulated by the Blitar Regent Regulation No. 127 of 2022. This study uses interviews, observations, and document analysis to explore the apply digital technology in improving health services, including its effectiveness, challenges, and opportunities, with the aim of compiling recommendations for further development in this field (Source: LKPJ 2024 Ngudi Waluyo Hospital).

The results of the interviews presented in Table 1 show the quality of public services at Ngudi Waluyo Wlingi Hospital based on the five dimensions of SERVQUAL, namely tangible, reliability, responsiveness, assurance, and empathy. In the tangible dimension, patients expressed their satisfaction with the physical facilities of the hospital such as cleanliness, comfortable waiting rooms, as well as the availability of modern medical equipment and special facilities such as isolation rooms and wheelchairs. In the reliability dimension, patient families and medical personnel revealed that inpatient services, responsive ambulances, and the existence of facilities such as Holter Heart and digital systems support the reliability of services. The responsiveness dimension is shown by ease of access through digital systems such as Anoman and Mobile National Health Insurance, transparency of room availability information, and the support of three internet providers that ensure the smooth operation of hospital IT. Meanwhile, the assurance dimension is reflected in a digital security system that protects patient data and complies with accreditation standards, strengthening public confidence in service quality. Finally, on the dimension of empathy, doctors in the emergency room explained the importance of personal communication with patients, including the use of regional languages such as Javanese, which shows sensitivity to cultural aspects in health services. Overall, the results of this interview reflect the improvement of the quality of public services at Ngudi Waluyo Hospital through the synergy among technological approaches and humanist values.

**Table 1:** Interview results based on the dimension of public service quality at Ngudi Waluyo Wlingi Hospital.

No	Dimension	Informants	Interview date	Interview contents
1	Tangible	Patient	13-Nov-24	Patients are comfortable with hospital facilities such as cleanliness, air-conditioned waiting rooms, comfortable chairs, and well-maintained environments.
			15-Nov-24	Patients appreciate modern facilities such as isolation rooms, wheelchairs, and adequate medical equipment for patients with respiratory conditions.
2	Reliability	Patient’s family	15-Nov-24	Inpatient facilities such as private bathrooms, sinks, and isolation rooms meet service standards, and ambulance services are quick to pick up emergency patients.
		Hospital staff	15-Nov-24	Medical personnel are highly qualified with continuous training, while facilities such as Holter Heart and digital systems improve the reliability of services.
3	Responsiveness	Hospital staff	16-Sep-24	The Anoman and Mobile JKN systems make it easier to register patients, while digital integration allows real-time access to medical results and patient health history.
		Administrative staff	16-Sep-24	Room availability information, queue numbers, and digital-based services provide transparency and efficiency in services, including quick access to patient data.
		IT staff	16-Sep-24	The use of three internet providers (In-Room, Iconet, Astinet) ensures smooth digital operations with a backup mechanism to avoid connection interruptions.
4	Insurance	Hospital staff	16-Nov-24	Digital security systems protect patients’ medical record data, support accreditation compliance, and provide holistic patient safety assurance.
5	Empathy	Emergency room doctor	18-Sep-24	Manual systems allow for more personalized interactions, but digitization increases efficiency without forgetting the importance of empathy for patient needs.
		Emergency room doctor	18-Sep-24	The use of regional languages such as Javanese creates personal closeness with patients, paying attention to cultural and social aspects in services.

Source: interview results, 2025.

Table 2 illustrates the results of interviews related to the quality of health services at Ngudi Waluyo Wlingi Hospital based on four main dimensions, namely user need, privacy and security, interoperability, and inclusion. On the user need dimension, IT staff explained that the digital system is developed based on user needs that are evaluated regularly through weekly meetings, as well as a trouble ticket system to record and follow up on complaints, while new features must be approved by the Medical Records Committee to maintain compliance with service standards. In privacy and security, the hospital shows a high commitment to the protection of patient data through server infrastructure that is not directly connected to the internet, strict access to server space, daily backup procedures, and the implementation of integrity pacts and employee oaths. The interoperability dimension can be seen from the integration of patient data with the national platform SatuSehat, the use of the Integrated Referral System application for the referral system, and coordination through the Health Office and Public Safety Center (PSC) even though they still face challenges in synchronization among agencies. Meanwhile, in the inclusion dimension, although staff from various units such as personnel, pharmacy, and hemodialysis admitted to being helped by the digital system, they realized the need for more intensive socialization, especially because the system was considered less friendly for elderly patients and Social Security participants who had difficulty accessing online services. Overall, these findings show that although the implementation of digitalization at Ngudi Waluyo Hospital has led to improved services, endeavors to improve integration, security, and inclusivity are still challenges that need to be addressed.

**Table 2:** Interview results based on the dimension of health service quality at Ngudi Waluyo Wlingi Hospital.

No	Dimension	Informants	Interview date	Interview contents
1	User need	The staff of the hospital are trying to get a hold of the	16 and 18 Sep 2024	Development of digital systems based on user needs through weekly evaluation meetings; the implementation of a trouble ticket system to record user complaints; The new feature requires the approval of the Medical Records Committee.
2	Privacy and security	The staff of the hospital are trying to get a hold of the	16 and 18 Sep 2024	The main server is not connected to the internet; access server space using fingerprints and manual locks; daily backups with two backup servers; Integrity pact and employee oath to maintain the confidentiality of patient data.
3	Interoperability	The staff of the hospital are trying to get a hold of the	17 and 18 Sep 2024	Data interconnection with the SatuSehat platform; the use of the Integrated Referral System application for patient referrals; The implementation of referrals is coordinated by the health office through the PSC, with the challenge of coordination among agencies.
4	Inclusion	Hospital Staff (Personnel, Hemodialysis, Pharmacy, Financial Administration) & Patients	15–19 Sep 2024	Staff found it helpful but had difficulty adapting to digital systems; requires more intensive socialization; digital systems are considered less simple for elderly patients; technical obstacles to online registration; Social Security patients need a more inclusive system.

Source: interview results, 2025.

The results of the interview research at Ngudi Waluyo Wlingi Hospital highlight the implementation of digital governance through four main dimensions (Table 3). The User Need dimension shows that the development of digital systems is oriented toward user needs, as evidenced by weekly evaluation meetings, a trouble ticketing system, and the approval of the Medical Records Committee for new features. Privacy and security are top priorities, with the implementation of servers without an internet connection, layered access using fingerprints, daily backups, and confidentiality commitments through integrity pacts and employee oaths. Data interoperability is facilitated through integration with the SatuSehat platform and the Integrated Referral System application, supporting data exchange among health facilities and referral coordination through the Health Office and PSC. However, the inclusion dimension shows that there are challenges in staff adaptation and ease of use for patients, especially the elderly and Social Security users, which requires more intensive socialization, a simpler interface, and improvements to technical constraints in online registration. These findings indicate that although Ngudi Waluyo Wlingi Hospital has made endeavors to implement digital governance, continuous endeavors are still needed to ensure inclusivity and ease of access for all users.

**Table 3:** Results of the four-dimensional digital governance interview at Ngudi Waluyo Wlingi Hospital.

No	Dimension	Informants	Interview date	Interview contents
1	User need	The staff of the hospital are trying to get a hold of the	16 and 18 Sep 2024	The system is developed according to the needs of the user through weekly meetings; complaints are recorded in the trouble ticket system; Medical Records Committee approval vital for new features
2	Privacy and security	The staff of the hospital are trying to get a hold of the	16 and 18 Sep 2024	Patient data is maintained with servers without an internet connection, fingerprint access, daily backups, backup servers, integrity pacts, and employee oaths to maintain confidentiality
3	Interoperability	The staff of the hospital are trying to get a hold of the	17 and 18 Sep 2024	Data integration through SatuSehat and Integrated Referral System supports data exchange among health facilities; the implementation of referrals is coordinated by the health office through the PSC
4	Inclusion	Staffing, hemodialysis, pharmacy, financial admin, and patient staff	15–19 Sep and 15 Nov 2024	The system helps but there are still adaptation constraints, limited socialization, complex interfaces, and technical constraints of online registration

Source: interview results, 2025.

Table 4 illustrates the results of interviews that examine the implementation of digital governance at Ngudi Waluyo Wlingi Hospital based on four main dimensions, namely user need, privacy and security, interoperability, and inclusion. On the user need dimension, IT staff explained that the development of the digital system is carried out in a responsive manner through the mechanism of weekly evaluation meetings and a trouble ticket system to accommodate user complaints, while the addition of new features must be approved by the Medical Records Committee, showing the existence of quality control and user participation in the innovation process. In the privacy and security dimension, patient data is strictly guarded using servers that are not connected to the internet, fingerprint-based server room access, daily backup systems, and the implementation of integrity pacts and employee oaths, indicating a high commitment to data protection and regulatory compliance. For the interoperability dimension, the hospital system has been integrated with national platforms such as SatuSehat and Integrated Referral System in supporting the exchange of patient data among health care facilities, although the implementation of referrals requires cross-agency coordination through the PSC. Meanwhile, in the aspect of inclusion, digitalization has indeed benefited staff in various units, but there are still obstacles in user adaptation, low digital literacy, complexity of interface design, and technical obstacles in the online registration process, especially for elderly patients and Social Security participants. Overall, this data shows that digital governance practices at Ngudi Waluyo Hospital have developed structurally and technically, but still require improvements in inclusivity and ease of access for all user groups.

**Table 4:** Interview results based on the digital governance dimension at Ngudi Waluyo Wlingi Hospital.

No	Dimension	Informants	Interview date	Interview contents
1	User need	The staff of the hospital are trying to get a hold of the	16 and 18 Sep 2024	The digital system is developed based on user needs through weekly evaluation meetings and a trouble ticket system to record complaints; New features need approval from the Medical Records Committee
2	Privacy and security	The staff of the hospital are trying to get a hold of the	16 and 18 Sep 2024	Patient data is protected with servers without an internet connection, fingerprint access, daily backups, backup servers, as well as integrity pacts and employee oaths to maintain confidentiality
3	Interoperability	The staff of the hospital are trying to get a hold of the	17 and 18 Sep 2024	The digital system uses the SatuSehat and Integrated Referral System platforms for data integration among health facilities; the implementation of referrals is coordinated by the health office through the PSC
4	Inclusion	Staffing, hemodialysis, pharmacy, financial admin, and patient staff	15–19 Sep and 15 Nov 2024	Digitalization has helped but there are still adaptation constraints, low digital literacy, complex interface design, and technical problems with online registration that hinder access for Social Security patients and the elderly

Source: interview results, 2025.

Table 5 presents the results of interviews regarding the implementation of good governance principles in public services at Ngudi Waluyo Wlingi Hospital, focusing on the dimensions of efficiency, transparency, service quality, and community participation. In efficiency, Arjuna Pharmaceutical Depot innovation with the “No Queue” system has succeeded in reducing the waiting time for medication collect a maximum of 8 min for simple prescriptions, which significantly improves the convenience and speed of pharmaceutical services. In the transparency dimension, the hospital actively publishes programs and services through websites and social media, provides free birth services along with administrative documents such as deeds, Id Card, and family members free from charge, and opens access to financial information in public spaces and digitally, although it still faces technical obstacles. Internal transparency is also improved through a water-cooling system that allows employees to submit complaints anonymously for organizational evaluation. In service quality, the public satisfaction survey showed an increase, supported by the use of social media as an open aspiration channel; patients and their families express their appreciation for fast, clean, friendly, and fair service, without discrimination among Social Security patients and the general public. Finally, in the dimension of community participation, RSUD routinely holds public dialogue and service socialization, building two-way communication with the public and the media to support participation-centered governance. These findings show that Ngudi Waluyo Hospital has applied good governance principles in improving transparency, efficiency, and quality of public services by actively involving the community.

**Table 5:** Interview results based on the principles of good governance in public service of Ngudi Waluyo Wlingi Hospital.

No.	Dimension	Informants	Interview date	Interview contents
1	Efficiency	The Board of Directors of the Ngudi Waluyo Hospital	16-Sep-24	Arjuna Pharmacy Depot *innovation* with the “No Queue” system reduces the waiting time for medicines to a maximum of 8 min for simple prescriptions, supporting the efficiency of pharmaceutical services and improving patient comfort.
2	Transparency	The Board of Directors of the Ngudi Waluyo Hospital	16-Sep-24	The hospital publishes services and innovations through its website and social media, including free shuttle services to improve patient comfort and accessibility.
3	Transparency	The Board of Directors of the Ngudi Waluyo Hospital	16-Sep-24	The hospital provides free childbirth services as well as birth certificates, ID CARD, and KK free from charge. Financial information is posted in public spaces and is also available digitally, although there are still technical constraints.
4	Transparency	The Board of Directors of the Ngudi Waluyo Hospital	16-Sep-24	Water *cooling* systems allow employees to submit complaints anonymously; used as internal evaluation materials to improve overall organizational transparency.
5	Quality of service	Deputy Director of Ngudi Waluyo Hospital	16-Sep-24	Public satisfaction surveys show an improvement despite potential bias; Social media is used as a validation and a forum for open complaints from patients.
6	Quality of service	Patients and patients’ families	15–16 Nov 2024	Patients appreciate the friendliness, promptness, cleanliness, and fairness of the service without discrimination among Social Security patients and the general public; The service is considered fast and professional.
7	Community participation	Public relations team of Ngudi Waluyo Hospital	15–16 Nov 2024	RSUD routinely holds public dialogues and service socialization with the media and the public to receive input, explain innovations, and build two-way communication within the framework of *people-centered governance*.

Source: interview results, 2025.

The findings from all tables show that Ngudi Waluyo Wlingi Hospital has made significant progress in improving the quality of public and health services through the implementation of digitalization and good governance principles. Service innovations, such as the “No Queue” system at the Pharmacy Depot and the use of digital platforms such as Anoman, Mobile National Health Insurance, SatuSehat, and Integrated Referral System, have increased the efficiency, transparency, and accessibility of services. Digital systems are developed based on user needs with strong data security, reflecting the implementation of structured digital governance. However, challenges are still found, especially in inclusivity, limitations of digital literacy, complexity of interfaces, and technical obstacles felt by elderly patients and Social Security users. In addition, although surveys and public communication channels have been used to improve the quality of services, staff adaptation to digital systems still requires support in the form of further training and socialization. Overall, Ngudi Waluyo Hospital has shown a strong commitment to digital transformation and participatory governance, but it is necessary to continue to strengthen system integration, strengthening human resources, and service inclusivity so that digitalization endeavors can run more optimally and equitably.

## 5 Discussion

The results of field findings through interviews, observations, and document studies regarding the implementation of digital governance at Ngudi Waluyo Wlingi Hospital. The goal is to relate empirical reality with the theory of public administration and digital-based health service management. The main focus of the discussion was how digitalization contributes to improving service quality and how the digital governance approach is used as an analytical framework to identify the achievements, obstacles, and challenges of hospital digital policies. In this context, Ngudi Waluyo Wlingi Hospital is positioned not only as a medical service provider, but also as a strategic implementer of public policies in the health sector.

**Figure 1:**
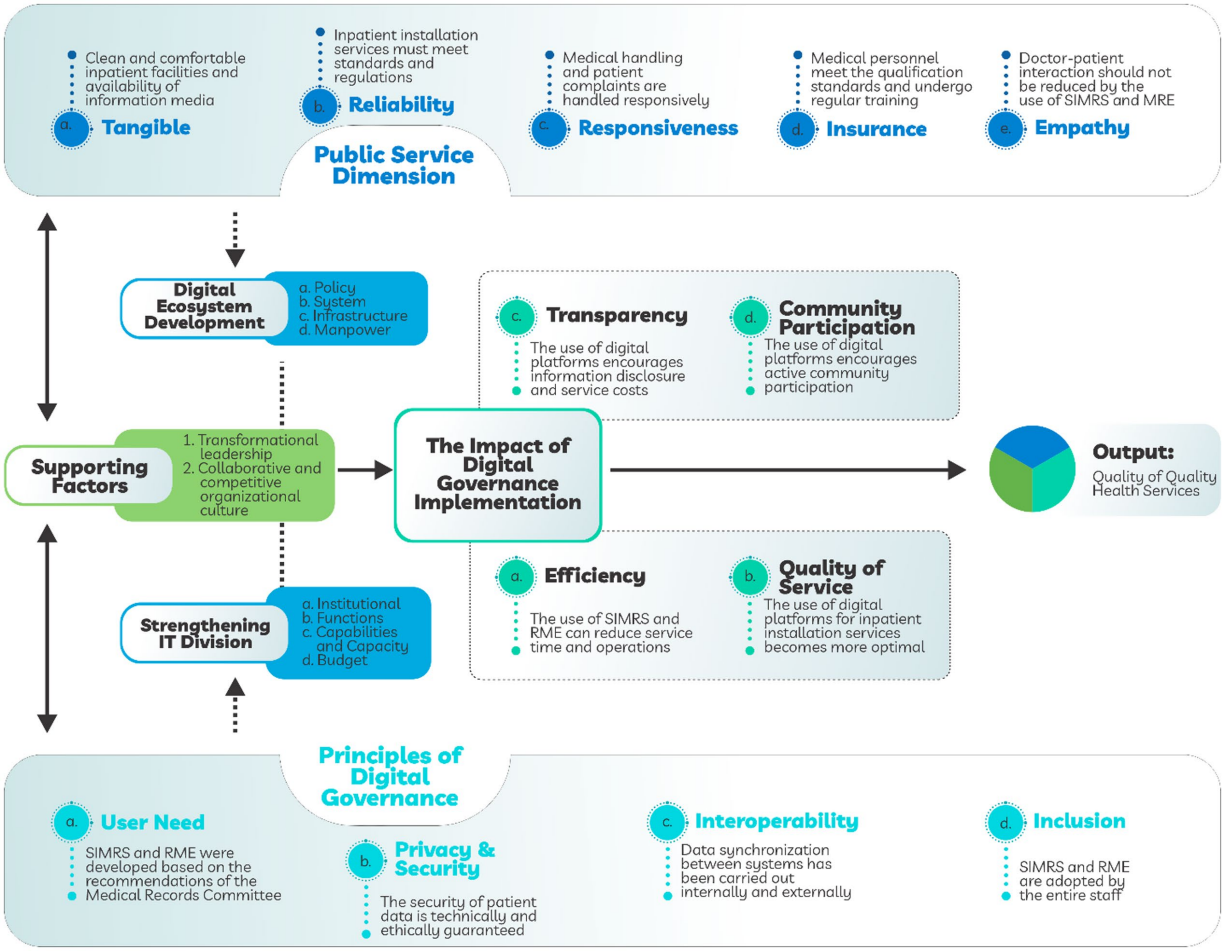
Model of the framework for the impact of digital governance implementation on the quality of health services.

Figure 1 illustrates a conceptual framework model on the impact of the implementation of digital governance on the quality of health services at Ngudi Waluyo Wlingi Hospital, which is structured through four main components: public service dimensions, digital governance principles, supporting factors, and digital ecosystem development. Analysis of the dimensions of public service quality at Ngudi Waluyo Wlingi Hospital shows that the integration among digitalization and good governance can strengthen patients’ perception and experience of service quality.

In the tangible dimension, the improvement of physical facilities such as comfortable treatment rooms and the use of digital information media has given the impression of professionalism and transparency, in line with the findings of Mattei (4) who stated that digital governance can strengthen the perception of service quality through data-based and accountable infrastructure. The reliability dimension is reflected in compliance with service protocols, diagnostic accuracy, and the use of digital systems for interoperably integrated patient data management. This is reinforced by the study of Reale et al. (20) which shows that a national interoperability system based on AI and legal data governance significantly improves diagnostic reliability and continuity of service.

Furthermore, in the responsiveness dimension, the speed of response to patient needs and complaints is increased through the digitization of service processes such as EHR and hospital management information systems. However, Flaherty (21) emphasized the importance of an ethical and participatory digital governance framework for systems to remain efficient and humane in responding to patient needs. For the assurance dimension, the capacity and competence of human resources are honed through a digital-based training system and data-based quality monitoring, by the findings of Said (22) which shows that the apply big data analytics can increase accountability and quality of health services systemically. Meanwhile, in the empathy dimension, although digitalization has the potential to reduce direct interaction, approaches based on local wisdom such as the use of regional languages have proven to be able to maintain a personal touch in service. These findings are in line with Raeside (23), which emphasizes the importance of adapting digital approaches to local cultures and specific age groups to maintain emotional closeness among patients and service providers. Thus, these five dimensions show that digital governance applied in an adaptive and inclusive manner can support the improvement of the quality of hospital public services without ignoring the humanistic aspect of health services.

The principles of digital governance in the management of health services at Ngudi Waluyo Wlingi Hospital include four main elements: user need, privacy & security, interoperability, and inclusion. Each of these principles plays a key role in ensuring that Hospital Management Information Systems (HIMS) and RMEs function optimally in supporting efficient, safe, and equitable services. The principle of user need is reflected in the development of HMIS and RME which are based on the recommendations of the Medical Records Committee, showing the adaptation of the system to clinical and administrative operational needs. This is in line with Fennelly et al. (24), who affirmed that the participation of users (physicians and healthcare workers) in EHR design is a key factor in the success of national implementation.

In privacy & security, digital systems are designed to maintain the confidentiality of patient data technically and ethically. Research by Ambalavanan et al. (25) identified that data security and proper system architecture are critical elements in the overall integration of health data. Interoperability enables data synchronization among internal and external systems, supporting real-time data-driven clinical decision-making. This is reinforced by Lam et al. (26) who stated that interoperability is a prerequisite for the success of digital smart health and aging care systems. The HL7 FHIR standard and cross-platform semantic integration are the main challenges that must be addressed to achieve full interoperability (25).

The principle of inclusion is affirmed through the adoption of the system by all hospital staff, including training and technical support so that no work unit is left behind in digital transformation. This is in line with the analysis of Supangkat et al. (27), which states that digital inclusion in public digital identity systems must take into account inequalities in technical capabilities, digital literacy, and organizational readiness. In addition to core principles, the success of digital governance is highly dependent on the organization’s supporting factors, namely transformational leadership and collaborative work culture. Ray (28) said that the integration of Web3 and AI technology in service systems will only be effective if supported by organizational governance that is open, adaptive, and responsive to technological changes. By combining the principles of digital governance and organizational supporting factors, Ngudi Waluyo Wlingi Hospital creates a strong foundation to build an inclusive, adaptive, and patient-oriented healthcare digital ecosystem, in line with global findings in digital health systems and public governance studies since 2020.

Strengthening the implementation of digital governance at Ngudi Waluyo Wlingi Hospital does not only depend on the apply basic principles such as transparency, efficiency, accountability, and participation, but is also supported by two key strategies that complement each other, namely strengthening the IT division and developing a sustainable digital ecosystem. Strengthening the IT division includes improving institutional aspects, work functions, increasing personnel capacity, and adequate budget allocation, as emphasized by Das (17) that institutional readiness and cross-functional coordination greatly determine the success of digital transformation in public services. Meanwhile, the development of the digital ecosystem is carried out through the formation of strong internal policies, the development of technological systems and infrastructure, and the increase in the capacity of human resources. This is in line with the findings of Hegazy and Mahboob (29) which emphasizes the importance of sustainable investment in smart city digital infrastructure and active community involvement to strengthen technology-based services.

The implementation of digital governance at Ngudi Waluyo Hospital has had a real impact on four main aspects of service. First, transparency is improved through online disclosure of service and cost information, strengthening accountability and encouraging public participation as reflected in the Local Governance Performance Index study (30). Second, the efficiency aspect can be seen from the optimization of HMIS and RME which are able to cut service time, similar to the success of TraceTogether in Singapore which combines operational efficiency and public trust (31). Third, the quality-of-service aspect has improved through the integration of digital tools that accelerate the service process and improve relationships among internal units, by the views of Abdullayev et al. (32) who emphasize the role of digital technology in boosting the quality-of-service interactions.

Furthermore, the use of advanced technologies such as AI and interoperability systems is also a driver of digital governance acceleration. This is supported by research (33) which shows that AI plays an important role in building smart healthcare and smart governance that is responsive to the needs of digital citizens. Thus, the strategy of strengthening IT institutions and developing a mature digital ecosystem has created a more transparent, efficient, quality, and participatory healthcare environment. All of these aspects synergistically encourage the improvement of the quality of hospital public services that are more adaptive to technological developments and the needs of modern society.

## 6 Conclusion

This research aims to explore how digital governance is implemented at Ngudi Waluyo Wlingi Hospital and its contribution to improving the quality of public health services. The findings show that the integration of digital systems—such as HMIS, RME, SatuSehat, and Integrated Referral System—has had a positive impact on various dimensions of service quality, namely efficiency, transparency, service reliability, patient satisfaction, and responsiveness. The apply the four main principles of digital governance—user need, privacy & security, interoperability, and inclusion—is carried out in an adaptive and structured manner. This system has simplified administrative processes, accelerated access to information, and improved medical decision-making, while maintaining cultural approaches such as the use of regional languages to strengthen communication and empathy in service. The study also confirms that the success of digital transformation is not only determined by technology, but also by organizational readiness, including institutional strengthening of IT divisions, transformational leadership, and collaborative work culture. Ngudi Waluyo Wlingi Hospital shows that digital efficiency can go hand in hand with humanistic values, such as empathy and a personal approach to patients.

However, this study has limitations. The main challenge that is still faced is the aspect of digital inclusion, especially for elderly patients and SOCIAL SECURITY participants who experience obstacles in accessing and using the online system. Uneven digital literacy and complex interface design are also obstacles in the optimization of digital systems. Theoretically, these findings enrich the study of digital governance and digital health care systems by emphasizing the importance of integration among technology, participatory governance, and local context sensitivity. In practical terms, this study shows that public hospitals can significantly improve the quality of service through an inclusive and user-based digital transformation. Further research is recommended to evaluate the long-term impact of digitalization on patient health outcomes, cost efficiency, and the resilience of public service institutions, especially in rural or semi-urban areas. Comparative studies among regions or countries can also provide a broader understanding of effective and replicable digital transformation models. In addition, in-depth research on the development of digital systems that are friendly to vulnerable groups needs to be carried out so that digital innovation is truly inclusive and leaves no one behind.
